# Subclinical leaflet thrombus in patients with severe aortic stenosis and atrial fibrillation -ENRICH-AF TAVI study

**DOI:** 10.1038/s41598-024-65600-5

**Published:** 2024-06-28

**Authors:** Yasuhiro Otsuka, Masanobu Ishii, Noriaki Tabata, Seitaro Oda, Masafumi Kidoh, Yuichiro Shirahama, Koichi Egashira, Naoto Kuyama, Taku Rokutanda, Katsuo Noda, Eiji Horio, Tomohiro Sakamoto, Takashi Kudo, Hideki Shimomura, Tomokazu Ikemoto, Ryusuke Tsunoda, Taishi Nakamura, Kunihiko Matsui, Koichi Kaikita, Kenichi Tsujita, ENRICH AF TAVI Investigators

**Affiliations:** 1 Department of Cardiovascular Medicine, Graduate School of Medical Sciences, Kumamoto University, 1-1-1 Honjo Chuo-ku, Kumamoto, 860-8556 Japan; 2 Department of Medical Information Science, Graduate School of Medical Sciences, Kumamoto University, 1-1-1 Honjo Chuo-ku, Kumamoto, 860-8556 Japan; 3 Department of Diagnostic Radiology, Graduate School of Medical Sciences, Kumamoto University, Kumamoto, Japan; 4 Kumamoto Central Hospital, Kumamoto, Japan; 5 Saiseikai Kumamoto Hospital, Kumamoto, Japan; 6 Fukuoka Tokushukai Medical Center, Fukuoka, Japan; 7 Japanese Red Cross Kumamoto Hospital, Kumamoto, Japan; 8 Department of General Medicine and Primary Care, Kumamoto University Hospital, Kumamoto, Japan; 9 Division of Cardiovascular Medicine and Nephrology, Department of Internal Medicine, Faculty of Medicine, University of Miyazaki, Miyazaki, Japan

**Keywords:** Leaflet thrombosis, TAVI, DOAC, CFD, T-TAS, Computational biology and bioinformatics, Cardiology

## Abstract

Subclinical leaflet thrombosis (SLT) can be one of the causes of transcatheter heart valve (THV) failure after transcatheter aortic valve implantation (TAVI). We sought to clarify the formation process of SLT and thrombogenicity during the perioperative period of TAVI. This multicenter, prospective, single-arm interventional study enrolled 26 patients treated with edoxaban for atrial fibrillation and who underwent TAVI for severe aortic stenosis between September 2018 and September 2022. We investigated changes in maximal leaflet thickness detected by contrast-enhanced computed tomography between 1 week and 3 months after TAVI in 18 patients and measured the thrombogenicity by Total Thrombus-formation Analysis System (T-TAS) and flow stagnation volume by computational fluid dynamics (CFD) (n = 11). SLT was observed in 16.7% (3/18) at 1 week, but decreased to 5.9% (1/17) at 3 months after TAVI. Patients with SLT at 1 week had a significantly decreased maximal leaflet thickness compared to those without SLT. Thrombogenicity assessed by T-TAS decreased markedly at 1 week and tended to increase at 3 months. The stagnation volume assessed by CFD was positively associated with a higher maximum leaflet thickness. This study showed the course of leaflet thrombus formation and visualization of stagnation in neo-sinus of THV in the acute phase after TAVI.

## Introduction

Transcatheter aortic valve implantation (TAVI) is now widely accepted, even among patients with severe aortic valve stenosis (AS) classified as low risk^1^. However, as subclinical leaflet thrombosis (SLT) in transcatheter heart valves (THV) increases the risk of transient ischemic attack, stroke, and exacerbation of heart failure due to valve restriction, administering antithrombotic therapy during the TAVI perioperative period is critical^2–5^. Although current Japanese guidelines recommend dual antiplatelet therapy (DAPT) as antithrombotic therapy for TAVI, a previous report showed that patients receiving DAPT had about a 15% incidence of SLT^6^.

Severe AS can induce acquired von Willebrand disease, characterized by excessive uncoiling of high molecular weight (HMW) multimers of von Willebrand factor (vWF) due to intense shear stress at the valve stenotic region. These vWF-HMW multimers are then excessively cleaved and consumed by ADAMTS13, leading to decreased thrombogenicity. Because of the high risk of bleeding associated with acquired von Willebrand syndrome in severe AS, it is imperative to be cautious about bleeding complications during the perioperative period of TAVI^7^. Atrial fibrillation (AF) is prevalent in about 20% of patients undergoing TAVI, with both warfarin and direct oral anticoagulation (DOAC) widely used in those patients^8–10^. A previous study showed that patients treated with DOACs had a lower incidence of SLT, suggesting that anticoagulant therapy might be useful for managing leaflet thrombosis during the perioperative period of TAVI^6^. However, there are no definitive reports on which anticoagulant drugs are most effective, and the mechanisms and contributing factors of leaflet thrombosis have not been fully elucidated.

ENVISAGE-TAVI AF, was a multinational, prospective, randomized trial that compared the efficacy and safety of edoxaban with that of vitamin K antagonists in patients undergoing TAVI. The trial demonstrated that the efficacy of edoxaban was noninferior to that of vitamin K antagonists. However, it also found a higher incidence of major bleeding with edoxaban than with vitamin K antagonists^11^. A sub-analysis of the Japanese population, however, indicated that edoxaban and VKA treatment exhibited similar safety and efficacy^12^. To further investigate the formation process of SLT on THV and its thrombogenicity during the perioperative period of TAVI, we conducted the ENRICH-AF TAVI (Edoxaban Regimen for the Prevention of Subclinical Valve Leaflet Thrombosis in Patients with Atrial Fibrillation Following Transcatheter Aortic Valve Implantation) study. We compared changes in maximal leaflet thickness at 1 week and 3 months post-TAVI in severe AS patients with AF treated with edoxaban. In addition, we analyzed thrombus formation using computational fluid dynamics (CFD) and the Total Thrombus-formation Analysis System (T-TAS). Furthermore, we identified factors related to maximal leaflet thickness, thereby clarifying the pathogenesis and contributing factors of SLT.

## Methods

### Study design and participants

All procedures were conducted in accordance with the Declaration of Helsinki and its amendments. This study received approval from the Human Ethics Review Committee of Kumamoto University Hospital, Japan. Written informed consent was obtained from each participant. This study is registered with the unique identifier jRCTs071180025 at https://rctportal.niph.go.jp, and received financial support from the Daiichi Sankyo Co., Ltd.

This multicenter, prospective, single-arm interventional study enrolled 26 consecutive patients with severe AS who underwent TAVI between September 2018 and September 2022 at several institutions, including Kumamoto University Hospital, Kumamoto Central Hospital, Saiseikai Kumamoto Hospital, Fukuoka Tokushukai Medical Center, and Japanese Red Cross Kumamoto Hospital. Details on sample size calculation are provided in the Methods section of the Supplemental material. The inclusion criteria were: (1) severe symptomatic AS, (2) scheduled to undergo TAVI, and (3) prevalent AF requiring oral anticoagulant therapy. The following exclusion criteria were applied: (1) patients with contraindications for edoxaban, (2) complicated by valvular AF due to rheumatic mitral valve stenosis or post mechanical valve replacement, and (3) patients who are judged inappropriate for study participation by a physician. Based on preliminary sample size calculations (Methods section of the Supplemental material), we aimed to enroll 98 patients, however, due to COVID-related enrollment issues, only 26 patients were ultimately included (Supplementary Fig. 1).

The multidisciplinary heart team responsible for performing TAVI assessed patient eligibility, selected the appropriate THV[SAPIEN 3 (Edwards Lifesciences, Irvine, CA) or Evolut R/PRO/PRO + (Medtronic, Minneapolis, MN)], and determined the suitable access site. For the transfemoral approach, a cardiovascular surgeon performed both the surgical cut-down and closure. In cases where percutaneous femoral artery access was employed, vascular closure was achieved using Perclose ProGlide (Abbott Vascular Co, Abbott Park, IL). During the valve delivery, a 14Fr eSheath (Edwards Lifesciences) was used for SAPIEN 3 valves, while a 14–18 Fr InLine sheath (Medtronic) or an 18–22 Fr GORE DrySeal sheath was used for Evolut R/PRO/PRO + valves.

### Sampling points and antithrombotic regimen

All patients with AF received edoxaban prior to TAVI and continued its use unless they developed bleeding severe enough to warrant discontinuation. Edoxaban dosages were determined by patient characteristics: individuals weighing less than 60 kg were given a 30 mg tablet; those weighing 60 kg or more received a 60 mg tablet, with a possible reduction to 30 mg based on renal function (CrCl 30 ≤ and ≤ 50). For patients aged 80 and older, or those with a history of organ bleeding, low body weight, significantly reduced renal function (CrCl 15 ≤ and ≤ 30), daily NSAID use, or concurrent antiplatelet therapy, a reduced dose of 15 mg was administered. In the patients who underwent percutaneous coronary intervention prior to TAVI, single antiplatelet therapy (either aspirin 100 mg/day, clopidogrel 75 mg/day, or prasugrel 3.75 mg/day) in addition to edoxaban was administered. No other anticoagulant or antithrombotic drugs were used. Edoxaban was interrupted on the day of TAVI and resumed on the following day. For patients on anti-platelet therapy, the medication was generally continued throughout the TAVI procedure, except for those at high risk of bleeding, who may have their anti-platelet therapy discontinued at the discretion of the attending physician.

Blood samples were collected from the antecubital vein of each patient on admission or on the day of TAVI (baseline, edoxaban-free point), and at 1 week (trough point) and 3 months (trough point) post-TAVI. These samples were also used for T-TAS measurement and vWF-HMW multimer analysis, as detailed in the Supplementary Methods.

### T-TAS measurement

The T-TAS (Fujimori Kogyo Co., Tokyo, Japan) is a microchip-based flow chamber system designed to evaluate whole-blood thrombogenicity. It was developed as an easy-to-use system to quantitatively analyze thrombus formation^13^. Previous studies have demonstrated the usefulness of the T-TAS parameter in predicting 1 year bleeding events in patients undergoing PCI and a significant predictor of procedural bleeding events in patients undergoing catheter ablation for AF^14,15^. Briefly, this system analyzes different thrombus formation processes with a simple procedure using two microchips coated with different thrombogenic surfaces. The platelet chip (PL) is coated with type I collagen, facilitating platelet adhesion and aggregation, leading to capillary occlusion within the microchip. Conversely, the atheroma chip (AR) combines type I collagen with tissue thromboplastin, which activates platelets and initiates coagulation. Thrombus formation within these chips is assessed by monitoring changes in flow pressure. The area under the curve (AUC) for flow pressure is computed to assess platelet thrombogenicity inside the microchips. Specifically, the PL-AUC parameter represents the AUC for the first 10 min for the PL tested at a flow rate of 24 μL/min, while AR-AUC represents the AUC for the first 30 min for the AR tested at a flow rate of 10 μL/min.

### Assessment of THV leaflet thickness

To assess subclinical leaflet thrombus, defined as hypo-attenuated leaflet thickening (HALT) and reduced leaflet motion (RLM) in patients post-TAVI, contrast-enhanced computed tomography (CT) scans were performed at 1 week and 3 months following the procedure. All patients underwent electrocardiogram-gated CT with data acquisition of the aortic root at end-diastolic and end-systolic phase according to a site-specific protocol for CT imaging in assessment for post-TAVI in each institution. After all CT data was collected from each institution, certified radiologists at Kumamoto University Hospital, with masked access to patient's clinical and laboratory data, measured the maximal leaflet thickness. The diagnosis of leaflet thrombosis was based on the identification of HALT, along with the RLM, in one or more leaflet segments. These changes were verified in at least two distinct multiplanar reconstruction (MPR) images at both end-systole and end-diastole phases, with maximal leaflet thickness assessed on end-diastolic longitudinal MPR images. The specific location of the affected cusp was determined relative to the initial native cusp position and categorized as right, left, or non-coronary, as previously described^16^. Additionally, CT data obtained 1 week post-TAVI was used to calculate stagnation volume by CFD analysis, as detailed in Supplementary Methods and Supplementary Fig. 2.

### Endpoints

The primary endpoint was defined as the difference in maximal leaflet thickness measured between 1 week and 3 months post-TAVI. The secondary endpoints included (1) characteristics of subclinical valve leaflet thrombosis observed at 1 week and 3 months after TAVI, (2) changes in thrombus formation, assessed using the T-TAS and vWF-HMW multimers, measured before, 1 week, and 3 months post-TAVI, (3) stagnation volume computed by CFD analysis, (4) factors associated with the maximal leaflet thickness at 1 week post-TAVI, and the difference between 1 week and 3 months post-TAVI, and (5) the number of adverse events after TAVI, defined as a composite of all-cause death, myocardial infarction, stroke, TIA, bleeding complications, vascular complications, and the new onset of conduct disorder.

### Statistical analysis

Data were expressed as the median and interquartile range (IQR) for continuous variables, and as counts and percentages for categorical variables. Group comparisons were performed using the Mann–Whitney U test or the unpaired *t* test for continuous variables, and the Chi-square test or Fisher’s exact test for categorical variables, as appropriate. For the analysis of sequential observational data collected at baseline, and at 1 week and 3 months post-TAVI, mixed-effects models for repeated measures were used. Simple liner regression analyses were conducted to assess the maximum leaflet thickness at 1 week and the increase in maximum leaflet thickness between 1 week and 3 months post-TAVI. A two-sided *p* value < 0.05 was considered statistically significant. All statistical analyses were performed using R software version 4.0.5. (R Foundation for Statistical Computing, Vienna, Austria. https://www.R-project.org/.) and the Statistical Package for Social Sciences software version 23.0 (IBM Corporation, Armonk, NY).

## Results

### Patient characteristics

Of 26 severe AS patients with AF who underwent TAVI, 18 patients were finally analyzed in the contrast-enhanced CT assessment, as shown in Supplementary Fig. 1. Patient characteristics are summarized in Supplementary Table 1. The median age was 84.0 years (IQR: 79.8, 86.0), and 38% of the participants were male. The dosages of edoxaban administered were 60 mg/day for 1 patient (6.3%), 30 mg/day for 13 patients (81%), and 15 mg/day for 4 patients (22%). Differences in baseline characteristics between the patients treated with edoxaban 15 mg/day and 30/60 mg/day are summarized in Supplementary Table 2. Patients on the 15 mg/day dose were significantly older and had higher risk scores (EuroSCORE II and STS scores), lower levels of hemoglobin and platelet count, and did not receive anti-platelet therapy, compared to those on the 30/60 mg/day doses. Additionally, trough plasma concentrations of edoxaban 1 week post-TAVI were significantly lower in patients on the 15 mg/day dose (n = 4) compared to those on the 30 mg/day dose (n = 12), as shown in Supplementary Fig. 3.

### Difference in maximum leaflet thickness

**Figure 1:**
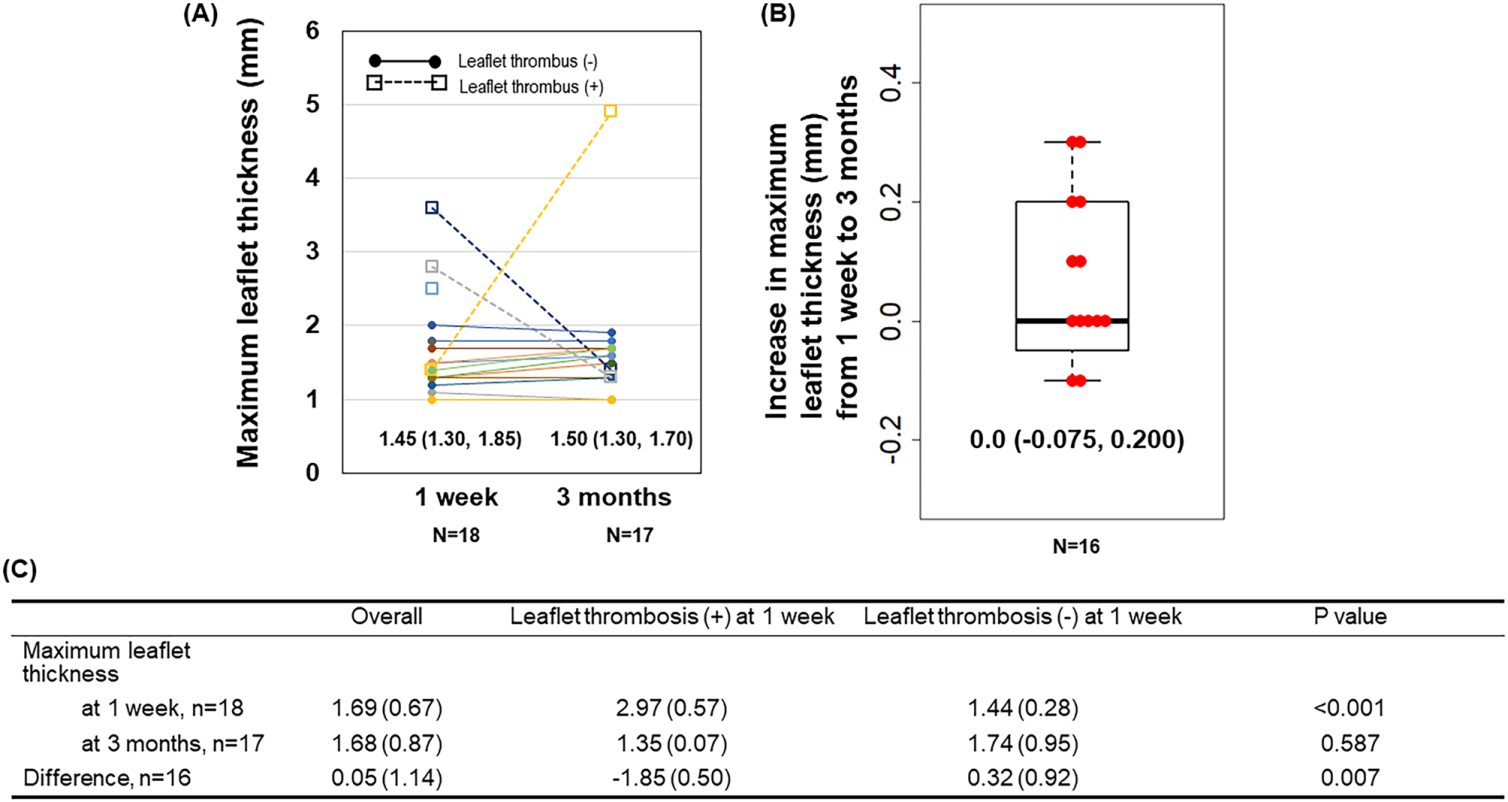
Difference in maximum leaflet thickness between 1 week and 3 months after TAVI stratified by leaflet thrombus. Maximum leaflet thickness was measured in 18 patients at 1 week and 17 at 3 months after TAVI (**A**). Box-and-whisker plot shows the increase in maximal leaflet thickness from 1 week to 3 months CT after TAVI (**B**). Table shows summary statistics of the maximal leaflet thickness at 1 week, 3 months, and the difference according to the presence of the leaflet thrombosis at 1 week after TAVI (**C**).

Maximum leaflet thickness was assessed by contrast-enhanced CT in 18 patients at 1 week and 17 patients at 3 months post-TAVI. One patient was lost to follow-up for CT assessment at 3 months due to incidence of cardiogenic cerebral embolism, and another was due to deterioration of renal function. Additionally, one patient did not have CT assessment at 1 week due to deterioration of renal function. Therefore, 16 patients received CT assessment at both 1 week and 3 months. The median maximum leaflet thickness was 1.45 mm (IQR: 1.30, 1.85) at 1 week and 1.50 mm (IQR: 1.30, 1.70) at 3 months post-TAVI (Fig. 1A). The median difference in maximum leaflet thickness between 1 week and 3 months post-TAVI was 0.0 mm (IQR: -0.075, 0.200), indicating no statistically significant change (Fig. 1B). SLT was observed in 3 (16.7%) patients without RLM at 1 week, and in 1 (5.9%) patient with 37.9% RLM at 3 months. An ad-hoc analysis showed that the maximum leaflet thickness at 3 months in patients with SLT at 1 week was significantly decreased compared to that in patients without SLT at 1 week (*p* = 0.007, Fig. 1C). The comparison of the increase in maximum leaflet thickness from 1 week to 3 months post-TAVI between patients who received antiplatelet therapy and those who did not revealed a significant decrease in thickness in the patients receiving antiplatelet therapy, as shown in Supplementary Fig. 4.

### Serial change in thrombogenicity

**Figure 2:**
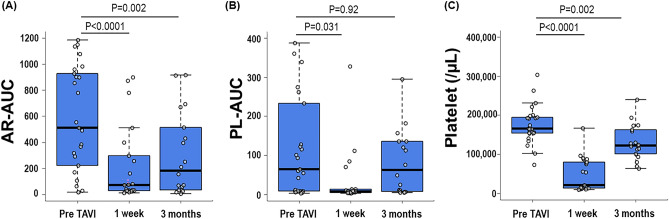
Changes in thrombogenicity parameters. These box-and whisker plots and each data point show sequential changes in AR-AUC (**A**), PL-AUC (**B**), and platelet count (**C**) before (n = 25) and at 1 week (n = 21) and 3 months (n = 18) after TAVI. AR-AUC indicates area under the curve for the atheroma chip; TAVI, transcatheter aortic valve implantation; PL-AUC, area under the curve for the platelet chip.

Various parameters related to thrombogenic activity were measured at baseline, 1 week and 3 months post-TAVI. As shown in Fig. 2, AR-AUC levels were significantly reduced at 1 week and 3 months post-TAVI, compared to baseline (*p* < 0.0001, *p* = 0.002, respectively). PL-AUC levels also decreased significantly at 1 week compared to the baseline (*p* = 0.031), but no significant change was observed at 3 months (*p* = 0.92). Platelet counts reached their lowest point at 1 week post-TAVI, increased by the 3 month mark, yet remained significantly lower than baseline values (Fig. 2C). The vWF-HMW multimer ratio tended to be higher at 1 week post-TAVI than baseline value, with not statistically significance (*p* = 0.07) (Supplementary Fig. 5). Subgroup analyses stratified by the combination with anti-platelet therapy and different dose of edoxaban showed that the combination with anti-platelet therapy and different dose of edoxaban were not associated with AR-AUC levels during the periprocedural period of TAVI (*p* = 0.405, *p* = 0.123, respectively) (Supplementary Fig. 6).

### Stagnation volume surrounding transcatheter heart valve

**Figure 3:**
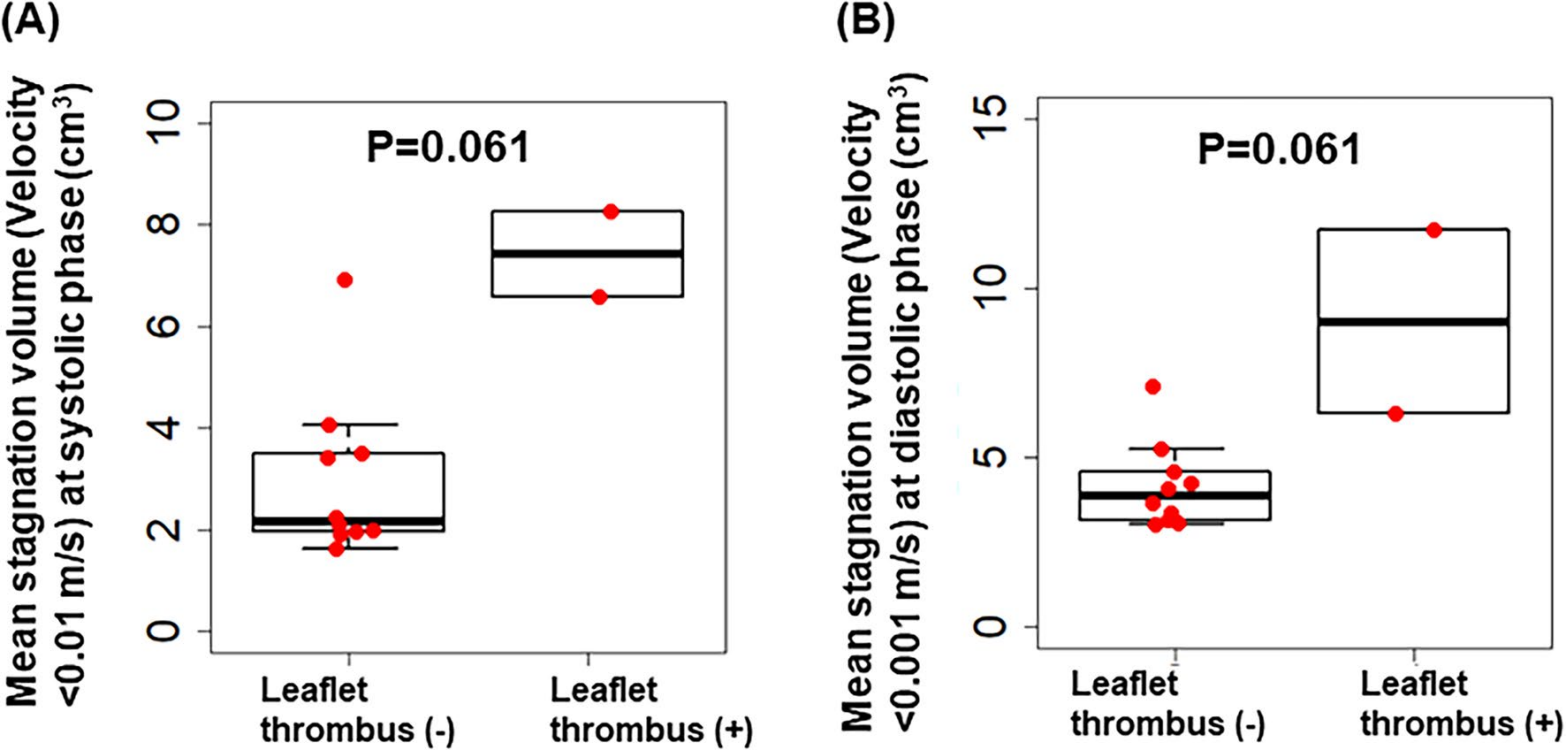
Difference in flow stagnation between patients with and without leaflet thrombus. Box-and-whisker plots show the difference in mean stagnation volume at systolic (**A**) and diastolic phase (**B**) between patients with and without leaflet thrombus at 1 week after TAVI.

CFD analysis to measure stagnation volume was performed using contrast-enhanced CT at 1 week post-TAVI in 11 patients. Of these, 2 patients were diagnosed with SLT, while 9 showed no signs of SLT. As shown in Fig. 3 and demonstrated in Videos 1 and 2, the mean stagnation volumes during both the systolic and diastolic phases tended to be higher in patients with SLT compared to those without, although these differences did not reach statistical significance (*p* = 0.061 for both phases). In the patients with SLT, flow stagnation in the neo-sinus and coronary cusp with leaflet thrombus during the systolic phase tended to be higher compared to those without (*p* = 0.099); during the diastolic phase, it was significantly higher compared to those without (*p* = 0.013), as shown in Supplementary Fig. 7.

### Factors related to leaflet thrombosis

To clarify the factors associated with maximum leaflet thickness at 1 week and the difference in leaflet thickness between at 1 week and 3 months post-TAVI, linear regression analyses were performed. Simple linear regression analysis showed that diabetes mellitus, pre-procedural AR III or IV, hemoglobin levels at 1 week, D-dimer levels at 1 week, AR-AUC levels at 1 week, and stagnation volume during both systole and diastole were positively associated with an increase in maximum leaflet thickness (Table 1). Regarding the difference in the maximum leaflet thickness between at 1 week and 3 months, age and a 15 mg/day dose of edoxaban were positively associated with an increased leaflet thickness at 3 months, while SLT incidence at 1 week was negatively associated (Table 2). The stagnation volume during diastole tended to be negatively associated with an increased leaflet thickness at 3 months, without statistically significance. Multivariable analysis could not be performed because of the limited sample size.

**Table 1:** Simple regression analysis for maximum leaflet thickness at 1 week after TAVI.

Variables	Maximum leaflet thickness at 1 week				
	Coefficient	SE	95% CI	Standardized coefficient	*P* value
Age, yrs	− 0.050	0.029	− 0.112, 0.011	− 0.399	0.101
Male	0.415	0.309	− 0.241, 1.071	0.318	0.198
Diabetes mellitus	0.847	0.380	0.041, 1.652	0.487	0.041
Chronic heart failure	− 0.035	0.326	− 0.726, 0.656	− 0.027	0.916
Edoxaban 15 mg/day	0.007	0.435	− 0.916, 0.929	0.004	0.988
Pre-procedural mean velocity, m/s	0.307	0.358	− 0.503, 1.117	0.858	0.413
Pre-procedural mean PG, mmHg	0.004	0.019	− 0.035, 0.043	0.052	0.839
Pre-procedural AR III or IV	0.894	0.285	0.290, 1.497	0.617	0.006
Hemoglobin, g/dL	0.030	0.094	− 0.170, 0.231	0.083	0.753
Platelet count, /µL, by 10,000	− 0.025	0.031	− 0.092, 0.042	− 0.201	0.440
Creatinine, mg/dL	0.816	0.739	− 0.759, 2.391	0.274	0.287
D-dimer, μg/mL	0.029	0.185	− 0.378, 0.435	0.047	0.880
AR-AUC by 100	0.026	0.046	− 0.071, 0.124	0.142	0.573
PL-AUC by 100	− 0.042	0.084	− 0.221, 0.136	− 0.125	0.622
vWF multimer index, by 1%	0.012	0.012	− 0.014, 0.038	0.253	0.328
Self-expanding valve type	− 0.421	0.417	− 1.310, 0.467	− 0.252	0.328
Valve size ≥ 26 mm	0.147	0.327	− 0.550, 0.844	0.115	0.659
Hemoglobin at 1 week	0.340	0.127	0.070, 0.610	0.570	0.017
Platelet count at 1 week, by 10,000	0.031	0.037	− 0.047, 0.110	0.213	0.411
Creatinine at 1 week	0.431	0.956	− 1.607, 2.469	0.116	0.659
D-dimer at 1 week	0.036	0.016	0.002, 0.069	0.532	0.041
AR-AUC at 1 week by 100	0.100	0.046	0.002, 0.199	0.488	0.047
PL-AUC at 1 week by 100	0.110	0.207	− 0.331, 0.552	0.136	0.602
vWF multimer index at 1 week, by 1%	0.003	0.010	− 0.019, 0.026	0.084	0.748
Mean PG at 1 week, mmHg	− 0.025	0.037	− 0.103, 0.054	− 0.170	0.513
Stagnation volume of velocity < 0.001 m/s during diastole	0.396	0.056	0.271, 0.521	0.913	< 0.001
Mean stagnation volume of velocity < 0.01 m/s during systole	0.182	0.076	0.013, 0.350	0.605	0.037

AUC indicates area under the curve for the atheroma chip; PL-AUC, area under the curve for the platelet chip; SE, standard error; CI, confidence interval; PG, pressure gradient; AR, aortic regurgitation; vWF, von Willebrand factor.

**Table 2:** Simple regression analysis for increase in maximum leaflet thickness from 1 week to 3 months CT after TAVI.

Variables	Increase in maximum leaflet thickness				
	Coefficient	SE	95% CI	Standardized coefficient	*P* value
Age, yrs	0.160	0.045	0.063, 0.257	0.687	0.003
Male	0.347	0.604	− 0.949, 1.642	0.152	0.575
Diabetes mellitus	− 1.314	0.823	− 3.079, 0.450	− 0.393	0.132
Chronic heart failure	0.300	0.586	− 0.957, 1.557	0.136	0.617
Edoxaban 15 mg/day	2.000	0.717	0.462, 3.538	0.598	0.014
Pre-procedural mean velocity, m/s	− 0.210	0.170	− 0.595, 0.174	− 0.381	0.247
Pre-procedural mean PG, mmHg	0.001	0.035	− 0.074, 0.076	0.008	0.975
Pre-procedural AR III or IV	− 1.149	0.559	− 2.349, 0.051	− 0.481	0.059
Hemoglobin, g/dL	− 0.117	0.181	− 0.507, 0.274	− 0.176	0.531
Platelet count, /µL, by 10,000	− 0.088	0.055	− 0.206, 0.030	− 0.407	0.132
Creatinine, mg/dL	− 1.228	1.345	− 4.133, 1.677	− 0.245	0.378
D-dimer, μg/mL	0.084	0.368	− 0.749, 0.918	0.076	0.824
AR-AUC, by 100	− 0.109	0.083	− 0.287, 0.069	− 0.331	0.210
PL-AUC, by 100	− 0.078	0.153	− 0.406, 0.249	− 0.136	0.615
vWF multimer index, by 1%	− 0.048	0.020	− 0.092, − 0.004	− 0.550	0.034
Self-expanding valve type	0.062	0.758	− 1.564, 1.687	0.022	0.936
Valve size ≥ 26 mm	0.400	0.582	− 0.848, 1.648	0.181	0.503
Platelet count at 1 week, by 10,000	− 0.026	0.067	− 0.169, 0.117	− 0.102	0.706
AR-AUC at 1 week, by 100	− 0.152	0.085	− 0.334, 0.029	− 0.433	0.094
PL-AUC at 1 week, by 100	− 0.050	0.376	− 0.856, 0.756	− 0.036	0.896
vWF multimer index at 1 week, by 1%	− 0.033	0.016	− 0.068, 0.003	− 0.486	0.066
Mean PG at 1 week, mmHg	− 0.062	− 0.256	− 0.198, 0.073	− 0.256	0.338
Platelet count at 3 months, by 10,000	− 0.061	0.065	− 0.201, 0.079	− 0.244	0.363
AR-AUC at 3 months, by 100	− 0.136	0.088	− 0.324, 0.053	− 0.382	0.145
PL-AUC at 3 months, by 100	0.012	0.117	− 0.238, 0.263	0.028	0.917
vWF multimer index at 3 months, by 1%	− 0.009	0.007	− 0.025, 0.008	− 0.329	0.272
Mean PG at 3 months, mmHg	− 0.086	0.057	− 0.210, 0.038	− 0.383	0.159
Stagnation volume of velocity < 0.001 m/s during diastole	− 0.409	0.212	− 0.881, 0.063	− 0.521	0.082
Mean stagnation volume of velocity < 0.01 m/s during systole	0.009	0.171	− 0.373, 0.391	0.017	0.957
Subclinical leaflet thrombosis at 1 week	− 2.171	0.681	− 3.631, − 0.712	− 0.649	0.007

AUC indicates area under the curve for the atheroma chip; PL-AUC, area under the curve for the platelet chip; SE, standard error; CI, confidence interval; PG, pressure gradient; AR, aortic regurgitation.; vWF, von Willebrand factor.

### Clinical outcomes

The details of clinical outcomes are summarized in Supplementary Results section and Supplementary Table 3.

## Discussion

The main findings of the ENRICH-AF TAVI study were as follows: (1) patient with SLT exhibited higher blood flow stagnation volumes; (2) a daily dose of 15 mg of edoxaban and a deficiency of vWF-HWM multimers were associated with an increase in leaflet thickness 3 months post-TAVI; (3) there was a significant association of preoperative AR concomitance, AR-AUC 1 week post-TAVI, and stagnation volume measured by CFD with the incidence of leaflet thrombosis 1 week post-TAVI. This study provided insights into the early stages of subclinical leaflet thrombus formation, changes in thrombogenicity measured by the T-TAS, and blood flow stagnation visualized by CFD analysis. These results offer valuable mechanistic insights into the development of SLT following TAVI.

Leaflet thrombosis is recognized as a critical mechanism of THV failure, leading to complications such as stroke, TIA, and exacerbation of HF due to restricted leaflet motion^2–6^. A previous observational study showed that the incidence of SLT was significantly higher in patients treated with DAPT at 14.9%, compared to 4% in those receiving anticoagulant therapy^6^. Furthermore, the incidence of SLT was comparable in patients with DOAC, compared to those with warfarin (3%, 4%, respectively)^6^. However, as the median time from aortic valve replacement to CT assessment was 83 days, the incidence of SLT in the acute phase after TAVI was unknown. Another study investigating early SLT after TAVI showed that 9.7% of patients on vitamin K antagonists exhibited leaflet thickening at median of 5 days post-TAVI^16^. Compared to this result, the frequency of SLT at 1 week post-TAVI observed in this study appears relatively high. Regarding later SLT, a significant decrease in leaflet restriction, thrombus extend, and maximum thickness was observed at follow-up CT performed at a median of 84 days in the anticoagulation group, whereas did not in the DAPT group^16^. This aligns with findings from the present study, where leaflet thickness in THV with leaflet thrombosis at 1 week decreased at 3 months post-TAVI. In the GALILEO-4D trial, a CT assessment at an average of 90 days post-TAVI revealed that at least one THV leaflet with grade 3 or higher motion reduction was observed in 2.1% of patients in the rivaroxaban group, compared to 10.9% in the DAPT group^17^. In addition, leaflet thickness of at least one leaflet was observed in 12.4% of the rivaroxaban and 32.4% of the antiplatelet group^17^. Despite some differences in participant backgrounds, the incidence of leaflet thickness at least one leaflet 3 months post-TAVI was lower at 5.9% in this study using edoxaban. Furthermore, the relationship between vWF-HMW multimers and leaflet thickness is shown in Tables 1 and 2. The results indicated no significant association between leaflet thickness 1 week post-TAVI and the vWF-HMW multimer index, as shown in Table 1; however, in Table 2, a lower pre-TAVI vWF multimer index was associated with a greater increase in leaflet thickness from 1 week to 3 months post-TAVI. One hypothesis suggests that patients with acquired von Willebrand disease, who typically exhibit a strong bleeding tendency, might experience an improvement in vWF-HMW multimers after TAVI. This improvement could lead to a compensatory shift towards hypercoagulability, potentially increasing thrombogenicity and resulting in thicker leaflets. Nevertheless, these findings are based on univariate analysis; thus, further investigation with a larger sample size is warranted to confirm these observations.

The results of this study, alongside findings from a previous study^16^., indicate that early leaflet thrombosis was found in a notable proportion of patients but tends to diminish over time. It was initially hypothesized that thrombogenicity would be enhanced during the early postoperative phase. However, contrary to these expectations, we observed a decrease in thrombogenicity, as shown in Fig. 2. In particular, postprocedural thrombocytopenia is a well-known phenomenon following TAVI^18,19^. This condition can be attributed to several mechanisms, such as inflammation, drug toxicity (e.g., heparin, aspirin or other antiplatelet drugs, warfarin, and direct oral anticoagulants), mechanical damage caused by shear stress (e.g., in the event of a paravalvular leak), activation of the coagulation cascade, a decrease in platelet production and impaired platelet renewal^18^. The transient decrease in platelet count following TAVI is associated with reduced thrombogenicity. Furthermore, findings from our previous study, the ATTRACTIVE-TTAS study, suggested that while vWF-HMW multimers improved soon after TAVI, both the AR-AUC levels and platelet counts were significantly lower 2 days post-TAVI, and then increased gradually^20^. Other previous studies on thrombocytopenia after TAVI have also showed a fall in platelet counts from 2 to 4 days following TAVI^21–23^. These observations suggest that there may be a rapid utilization of platelets and coagulation factors immediately post-TAVI, which temporarily reduces thrombus formation. This rapid depletion could then trigger a compensatory response, potentially leading to enhanced thrombus formation and contributing to the development of neo-sinus associated leaflet thrombus in THV. Supportive of this theory, regression analysis results shown in Table 1 indicate that higher AR-AUC levels at 1 week post-TAVI are positively associated with increased leaflet thickness, aligning with our hypothesis. In this study, subgroup analyses revealed that neither the combination with anti-platelet therapy nor different doses of edoxaban was associated with changes in the AR-AUC during the periprocedural period of TAVI (Supplementary Fig. 6). As shown in Fig. 2, platelet count was significantly associated with changes in the AR-AUC. Thus, the reason for decrease in the AR-AUC 3 months post-TAVI might be due to decrease in platelet count rather than to differences in antithrombotic therapy regimens. This observation aligns with findings from our previously reported ATTRACTIVE T-TAS study^20^. However, while antithrombotic therapies are thought to significantly impact AR-AUC, this study possibly failed to detect such associations, likely due to its small sample size and resultant low statistical power. Because the findings of this investigation remain hypothesis-generating, future hypothesis-testing study is needed.

Stasis or interrupted blood flow is one of the key components of Virchow’s triad^24^. Previous studies have provided mechanistic insight into the SLT formation of the THV by flow stagnation, identifying that risks of increased flow stasis of neo-sinus are THV expansion, implant depth, implant position, valve geometry and cardiac output^25–27^. However, these findings originated from in vitro flow studies, and no evidence has been established on directly investigating the association between leaflet thrombosis and flow stagnation within neo-sinus of THV using clinical patient data. To the best of our knowledge, this was the first in vivo study to evaluate this association. As shown in Table 1, simple regression analysis showed that increased flow stagnation was associated with leaflet thrombosis post-TAVI. However, as shown in Table 2, the increased flow stagnation was not associated with an increase in maximum leaflet thickness from 1 week to 3 months post-TAVI. Thus, the analysis warrants a more focused approach. This CFD analysis encompassed the entire aortic root region. A more targeted analysis specifically on the neo-sinus, rather than the entire aortic root, could potentially yield a more accurate estimation of the association between flow stagnation and leaflet thrombosis. Another important finding from the study is that simple linear regression identified a higher risk of increased leaflet thickness in the chronic phase associated with a 15 mg/day dose of edoxaban (Table 2). As shown in Supplementary Fig. 3, the plasma concentration of edoxaban at 15 mg/day was lower than at 30 mg/day, leading us to speculate that lower plasma concentrations may influence the increase in leaflet thickness. However, the use of plasma concentration as an explanatory variable in linear regression analysis was not feasible due to missing data; plasma concentrations were not measured in 5 out of the 16 subjects who underwent contrast-enhanced CT, resulting in more than 30% missing data. In addition, as shown in Supplementary Table 2, differences in patient characteristics between the 15 mg/day and 30/60 mg/day edoxaban suggest potential confounding factors that would require adjustment in a multivariate analysis. Larger studies are required to validate the results of this study and to further explore the mechanism of leaflet thrombosis.

### Limitations

This study has several limitations. First, the sample size was limited, particularly for primary endpoint analyses and multivariable analysis, which restricts the statistical power and, consequently, the reliability and generalizability of the findings. We initially planned this study including approximately 100 patients, but due to restrictions on hospital admissions for COVID-19 and fewer hospitalizations of patients undergoing TAVI with AF than anticipated, the actual sample size was significantly reduced. The statistically significant results observed in this study must be interpreted with caution due to the potential for these findings to be incidental, a concern attributed to the limited sample size. For example, a simple linear regression analysis showed that a daily dose of 15 mg edoxaban was positively associated with an increase in the maximum leaflet thickness, with statistically significance. However, this finding is based on a very small subset of only 4 patients treated with the 15 mg/day dosage of edoxaban. Such a small sample may not provide a robust basis for generalization, and there remains a significant possibility that the observed effect could be due to chance. Moreover, antithrombotic therapy was heterogeneous, with 3 different doses of edoxaban and 3 different combinations of antiplatelet agents. We initially planned to conduct a subgroup analysis to examine whether variations in these drug combinations influenced the formation of leaflet thrombosis in THV and the overall thrombogenicity. However, due to the small number of cases registered, it was not feasible to perform this analysis. To improve the detection power of future studies, it is crucial to standardize the variability in antithrombotic therapy regimens, enroll a larger number of patients, and ensure the reproducibility of the results. Second, the follow-up period after TAVI was limited up to 3 months in this study. A longer-term follow-up would provide clues to the clinical significance of leaflet thrombosis in prognosis after TAVI. Third, the assessment of maximum leaflet thickness and factors associated with leaflet thrombosis might not have accounted for other important risk factors such as age, comorbidities, and medication use. These factors could potentially influence the occurrence of leaflet thrombosis and maximum leaflet thickness. For the limited sample size, we could not adjust these factors in multivariable analysis. Fourth, due to the absence of a control group, the hypothesis that AF may be involved in the development of SLT following TAVI could not be investigated. Fifth, in the CFD analysis, patient-specific cardiac outputs and blood pressures were not used for boundary conditions. This decision was based on prior validation experiments by Coronary Flow Design, which demonstrated minimal differences between the outcomes using patient-specific data versus standard boundary conditions. Consequently, standard boundary conditions were applied in this study. However, this study did not conduct a direct comparison of the effects between patient-specific and standard boundary conditions in analyzing blood flow stagnation around the THV in the aortic root. Therefore, the potential biases introduced by this methodological choice remain unquantified, and their impact on the results cannot be definitively determined.

In conclusions, the ENRICH-AF TAVI study, a multicenter prospective single-arm observational study, demonstrated that the natural process of leaflet thrombus formation in the neo-sinus of THV and the dynamic fluctuations of thrombogenicity in the acute phase post-TAVI. These results suggest that blood flow stagnation may play a significant role in the development of leaflet thrombus.　Moreover, they highlight the challenge in suppressing thrombus formation with lower concentrations of anti-coagulants, particularly in patients requiring reduced doses of DOAC due to certain risk factors. However, due to the limited sample size, the potential of these results being incidental has not been conclusively eliminated. Therefore, larger future studies are necessary to validate these findings and further explore the mechanisms involved.

### Supplementary Information

Supplementary Information.

## Data Availability

The data underlying this article will be shared on reasonable request to the corresponding author.
