# Supplementary material for: Subclinical leaflet thrombus in patients with severe aortic stenosis and atrial fibrillation -ENRICH-AF TAVI study
Source: Sci Rep. 2024 Jun 28;14:14902. doi: 10.1038/s41598-024-65600-5 (PMC11213935; doi:10.1038/s41598-024-65600-5)
Supplement: Supplementary file 1 — Supplementary Information. [file 41598_2024_65600_MOESM1_ESM.pdf]

Supplemental material for Subclinical Leaflet Thrombus in Patients with Severe Aortic Stenosis and Atrial Fibrillation -ENRICH-AF TAVI study-

Authors: Otsuka Y et al.

TABLE OF CONTENTS

Supplementary Methods:

1. Assessment of vWF-HMW multimer
2. Measurement of plasma concentration of edoxaban
3. Stagnation volume assessed by CFD analysis
4. Sample size calculation

Supplementary Results:

1. Clinical outcomes

Supplementary Figure 1:

Study flowchart

Supplementary Figure 2:

Representative images and graphs of flow stagnation of aortic root after TAVI at systolic phase (A) and diastolic phase (B).

Supplementary Figure 3:

Plasma concentration of edoxaban at 1 week after TAVI

Supplementary Figure 4:

Increase in maximum leaflet thickness from 1 week to 3 months stratified by the combination with anti-platelet therapy

Supplementary Figure 5:

Changes in coagulation parameters

Supplementary Figure 6:

Changes in thrombogenicity parameters stratified by the combination with anti-platelet therapy and different dose of edoxaban.

Supplementary Figure 7:

Difference in flow stagnation between leaflets with and without thrombus

Supplementary Table 1:

Baseline characteristics of study population

Supplementary Table 2:

Baseline characteristics of patients with Edoxaban 15mg and 30/60mg

Supplementary Table 3:

Clinical outcomes in 24 patients

Supplementary Methods

1.Assessment of vWF-HMW multimer

To assess the change of vWF-HMW multimer during peri-procedural period, we performed vWF multimer analysis. A plasma sample from each patient was used for vWF multimer analysis (based on the established method of sodium dodecyl sulfate agarose gel electrophoresis [1]) outsourced to SRL, Inc. (Tokyo, Japan). The value of vWF-HMW multimers was estimated using Image J software and represented the relative amount of the largest multimers in the sample compared with those of the normal pooled plasma in the next lane of the same gel, and expressed as vWF-HMW multimer index (%). This index is defined as the ratio of the patients' vWF-HMW multimer ratio to the healthy control's ratio, as described previously [2,3].

2.Measurement of plasma concentration of edoxaban

Trough concentration of edoxaban at 1 week after TAVI was quantified by using the commercially available standardized assay kit (BIOPHEN DiXaI kit, HYPHEN BioMed, Paris, France), which is an anti-Xa chromogenic method for the *in vitro* quantitative determination of direct Factor Xa inhibitors in human plasma, according to the protocol supplied by the manufacturer.

3.Stagnation volume assessed by CFD analysis

CFD analysis can visualize blood flow condition and can quantify the flow streamline, flow velocity, WSS, oscillatory index, and other parameters, using a patient-specific 3D model based on the individual CT images. Using contrast-enhanced CT at 1 week post-TAVI, CFD analysis for measuring stagnation volume was conducted by Cardio Flow Design, Inc. (Tokyo, Japan), according to the method established previously [4,5,6,7]. Briefly, 3-dimensional patient-specific geometries of the area extending from the aortic root to the ascending aorta was reconstructed from the individual CT images. Computational meshes were generated by the ANSYS-ICEM 16.0 software (ANSYS Inc., Tokyo), and the finite volume solver package ANSYS Fluent 18.0 (ANSYS Inc.) was used to solve the Navier-Stokes equation of incompressible transient Newtonian fluid in setting. The

boundary condition was set as follows; mean aortic root inflow rate of 4,450mL/min, mean left coronary artery flow rate of 148 mL/min, mean right coronary artery flow rate of 74.2mL/min, and aortic root outflow pressure of 96.0 mmHg. The pulsatile flow was employed, and the stagnation volume of the aortic root with THV was calculated separately during systole and diastole [8,9]. Fluid-Structure Interaction analysis was not performed because it was not possible to calculate the movements of valves and blood vessel walls. Stagnation was defined as velocity <0.01 m/s at systole or velocity <0.001 m/s at diastole. The mean stagnation volume for the comparison of blood flow stagnation was calculated, as shown in the **Supplementary Figure 2**.

4.Sample size calculation

The primary endpoint of this study was the difference in maximal leaflet thickness between 1 week and 3 months after TAVI. For sample size calculation, the number of cases that can verify the difference in maximum leaflet thickness between 1 week and 3 months after TAVI should be registered. However, since there is no information regarding the maximum leaflet thickness in patients treated with edoxaban, the calculation should be based on the maximum leaflet thickness 1 week and 3 months after TAVI in patients treated with vitamin K antagonist phenprocoumon. Previous study showed that rate of patients with leaflet thickening in at least one valve leaflet was 9.7% in contrast-enhanced CT 5 days after TAVI, and the maximum leaflet thickness was 3.27mm at median 5 days and 0.7mm at 3 months after TAVI, with a standard deviation of 1.5mm for the difference [10].

Therefore, we set the following hypotheses: 1) the percentage of patients treated with edoxaban who exhibit valve leaflet thickening 1 week after TAVI was 9.7%. 2) the maximum leaflet thickness 1 week after TAVI in patients treated with edoxaban was 3.27mm. 3) The maximum leaflet thickness 3 months after TAVI in patients treated with edoxaban was decreased to 0.7mm. 4) the standard deviation of the difference between 1 week and 3 months after TAVI in patients treated with edoxaban was 1.5mm. At a power of 90% and a significance level of 5%, the required number of cases to verify the effect was 83 cases. Assuming a dropout rate of 15%, 98 cases were calculated as the required sample size for enrollment. However, our study faced recruitment difficulties due to the COVID-19 pandemic. We could not reach the target sample size of 98 patients, and we

terminated the study with 26 patients.

Supplementary Results

1. Clinical outcomes

Of 26 severe AS patients with AF who underwent TAVI, 24 patients were analyzed for clinical outcome assessment, as shown in **Supplementary Figure 1**. As shown in **Supplementary Table 2**, during the 1-week follow-up period, TAVI related complications were noted in 4 patients (ischemic stroke: n=1, major life-threatening or disabling bleeding complications: n=2, minor bleeding: n=1). During the 3-month follow-up period, clinical adverse events were recorded in 5 patients, including stroke (n=2), life-threatening or disabling bleeding (n=2) and minor bleeding (n=1). Both 2 cases of major bleeding and 2 cases of stroke were patients on edoxaban 30 mg/day.

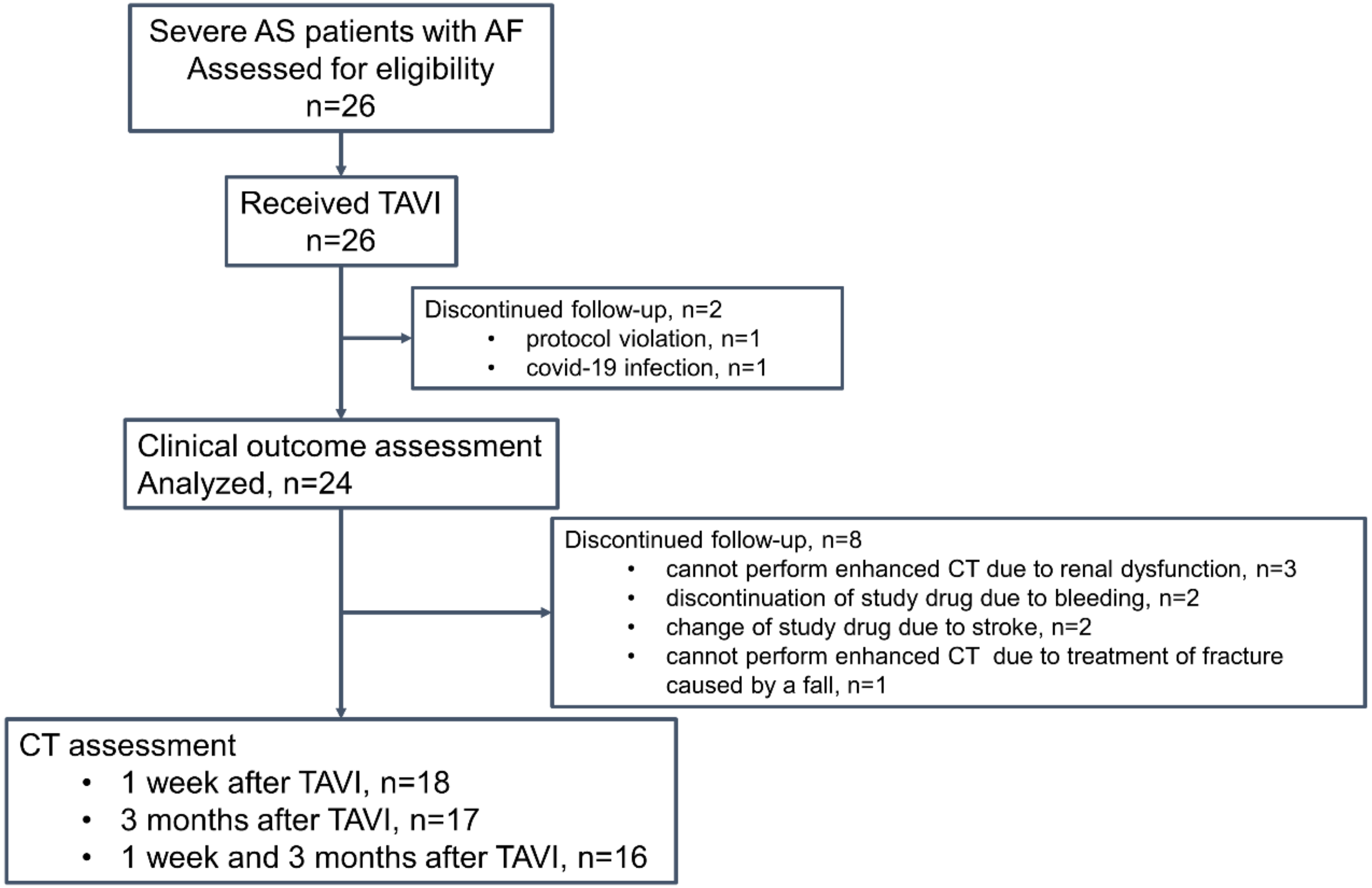

Supplementary Figure 1. Study flowchart.

AS indicates aortic stenosis; AF, atrial fibrillation; TAVI, transcatheter aortic valve implantation; CT, computed tomography.

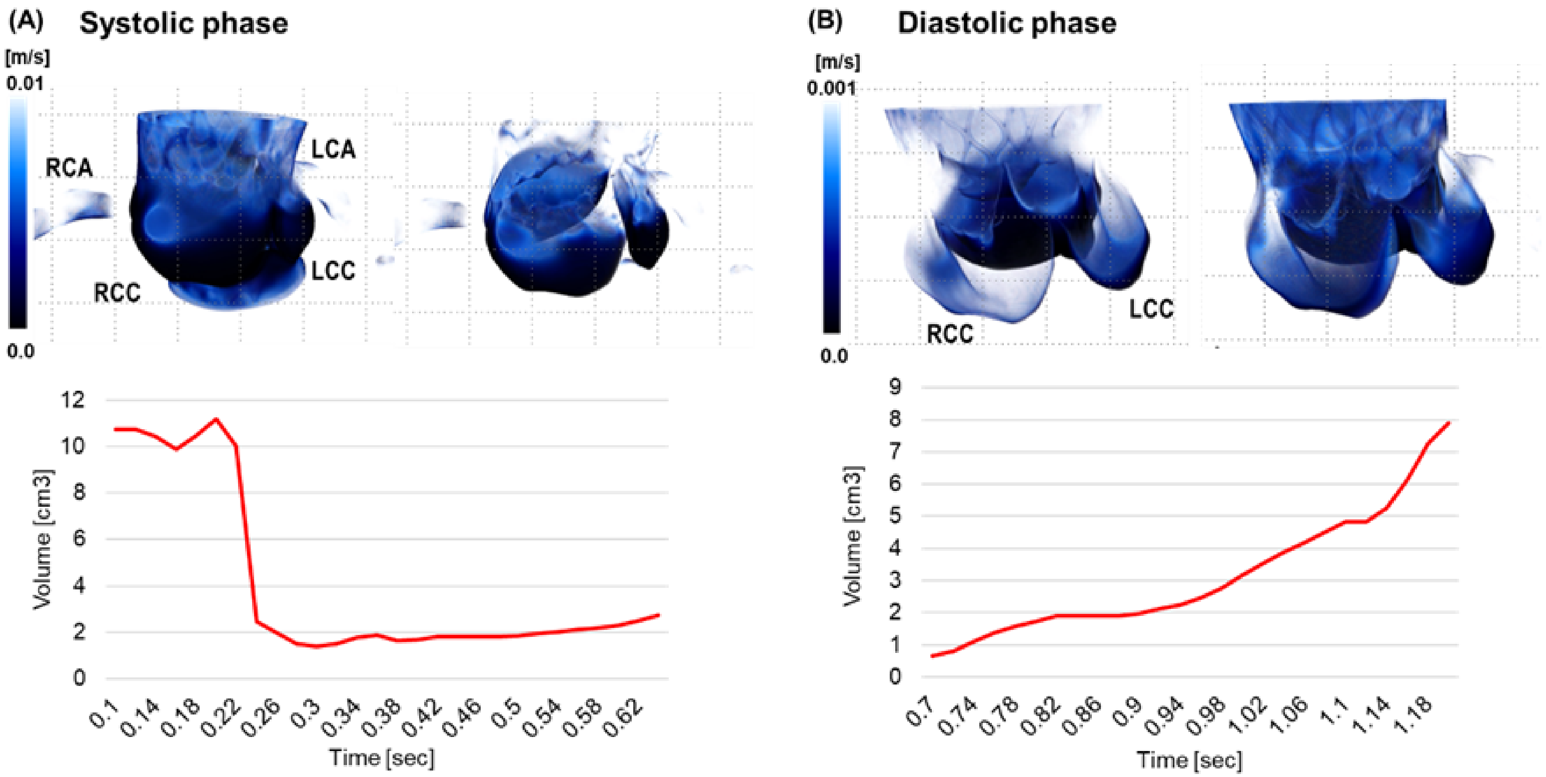

Supplementary Figure 2. Representative images and graphs of flow stagnation of aortic root after TAVI at systolic phase (A) and diastolic phase (B). Stagnation was defined as velocity <0.01 m/s at systole or velocity <0.001 m/s at diastole. The line graphs show the temporal changes in stagnation volume. The mean stagnation volume for the comparison of blood flow stagnation was calculated.

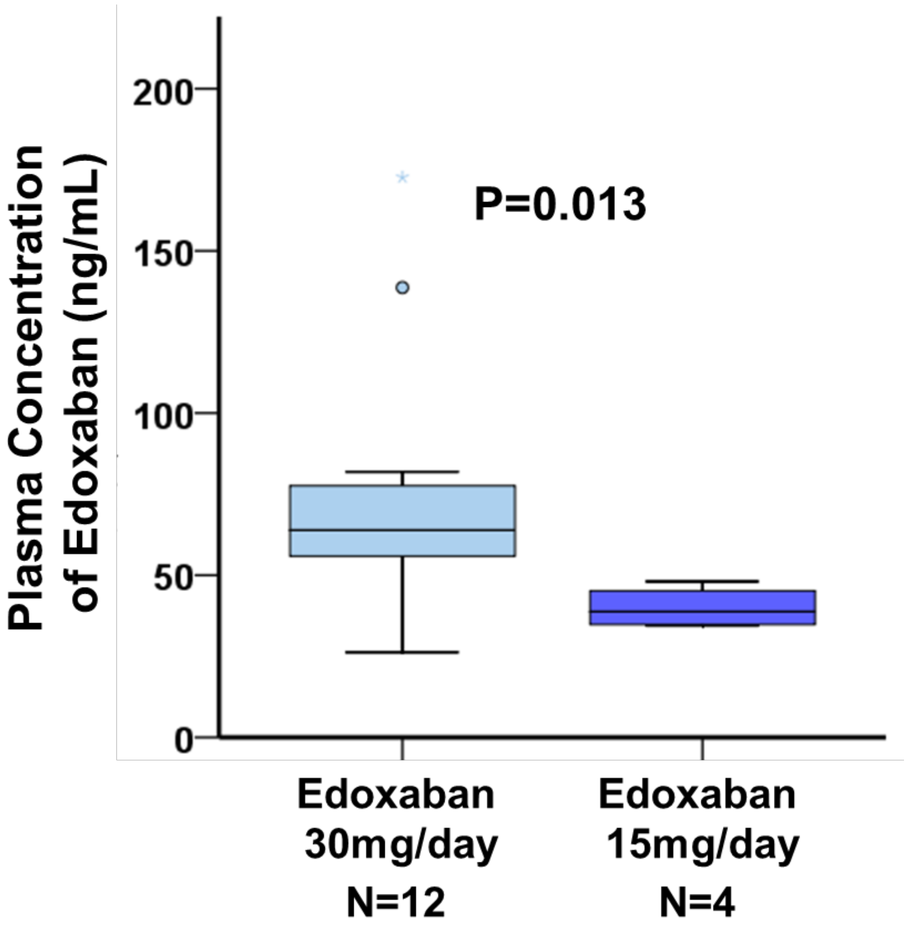

Supplementary Figure 3. Plasma concentration of edoxaban at 1 week after TAVI

These box-and whisker plots show plasma concentration of edoxaban in patients treated with 30 mg/day (n=12) and 15 mg/day (n=4) at 1 week after TAVI. One of the 13 patients treated with edoxaban 30 mg/day whose blood concentration was measured was excluded from the analysis because it was an outlier value of more than 300 ng/mL.

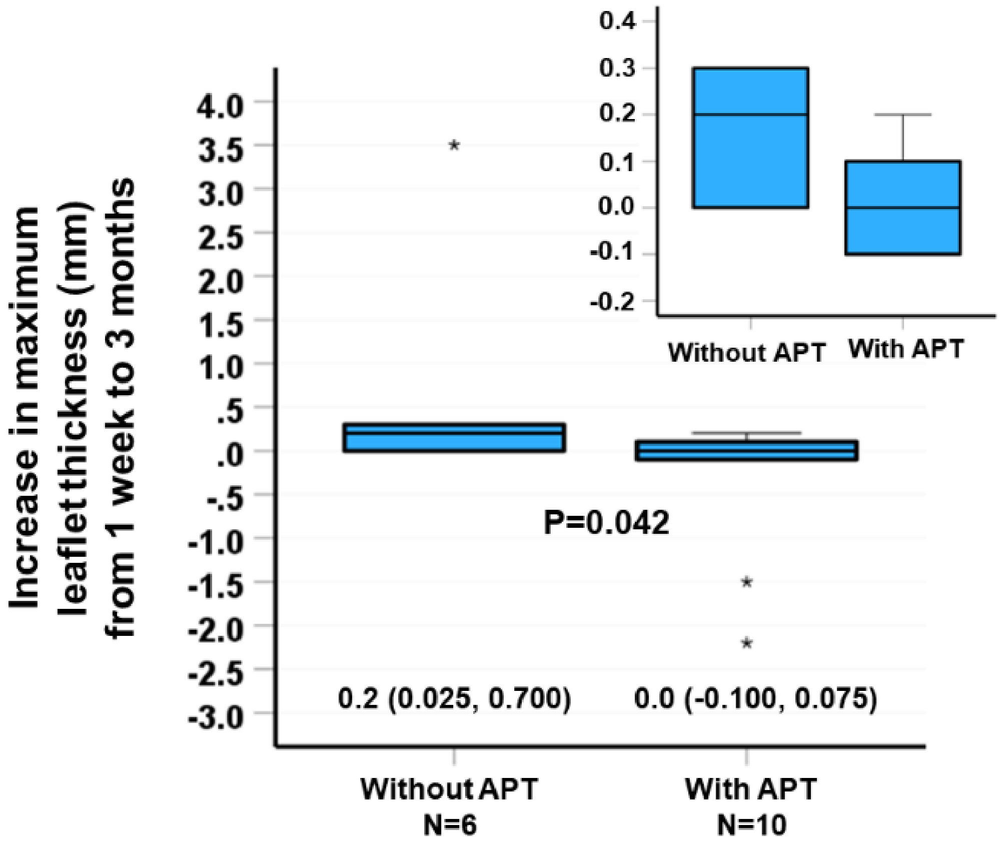

Supplementary Figure 4. Increase in maximum leaflet thickness from 1 week to 3 months stratified by the combination with anti-platelet therapy

The main panel displays the leaflet thickness at one week and three months post-TAVI in patients who received antiplatelet therapy and those who did not. The box in the upper right corner shows an enlargement of the main panel. APT indicates antiplatelet therapy: TAVI, transcatheter aortic valve implantation.

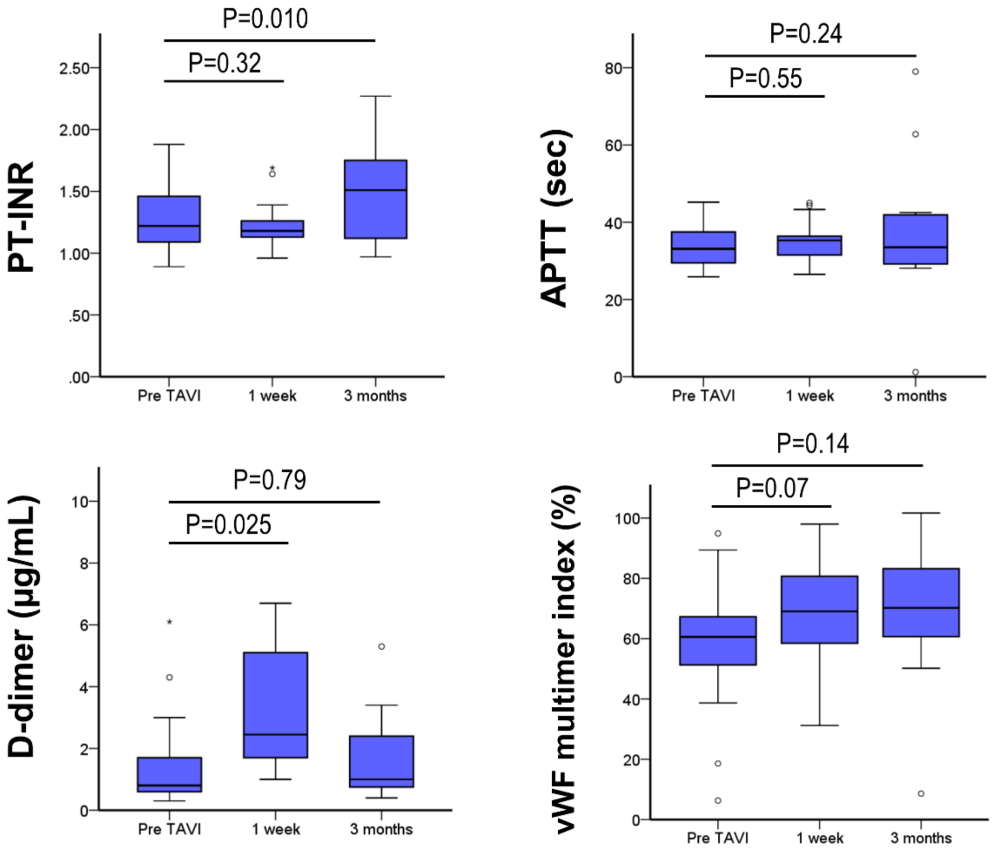

Supplementary Figure 5. Changes in coagulation parameters

These box-and whisker plots show sequential changes in PT-INR, APTT, D-dimer and vWF-HMW multimer index before and at 1 week and 3 months after TAVI. vWF indicates von Willebrand factor.

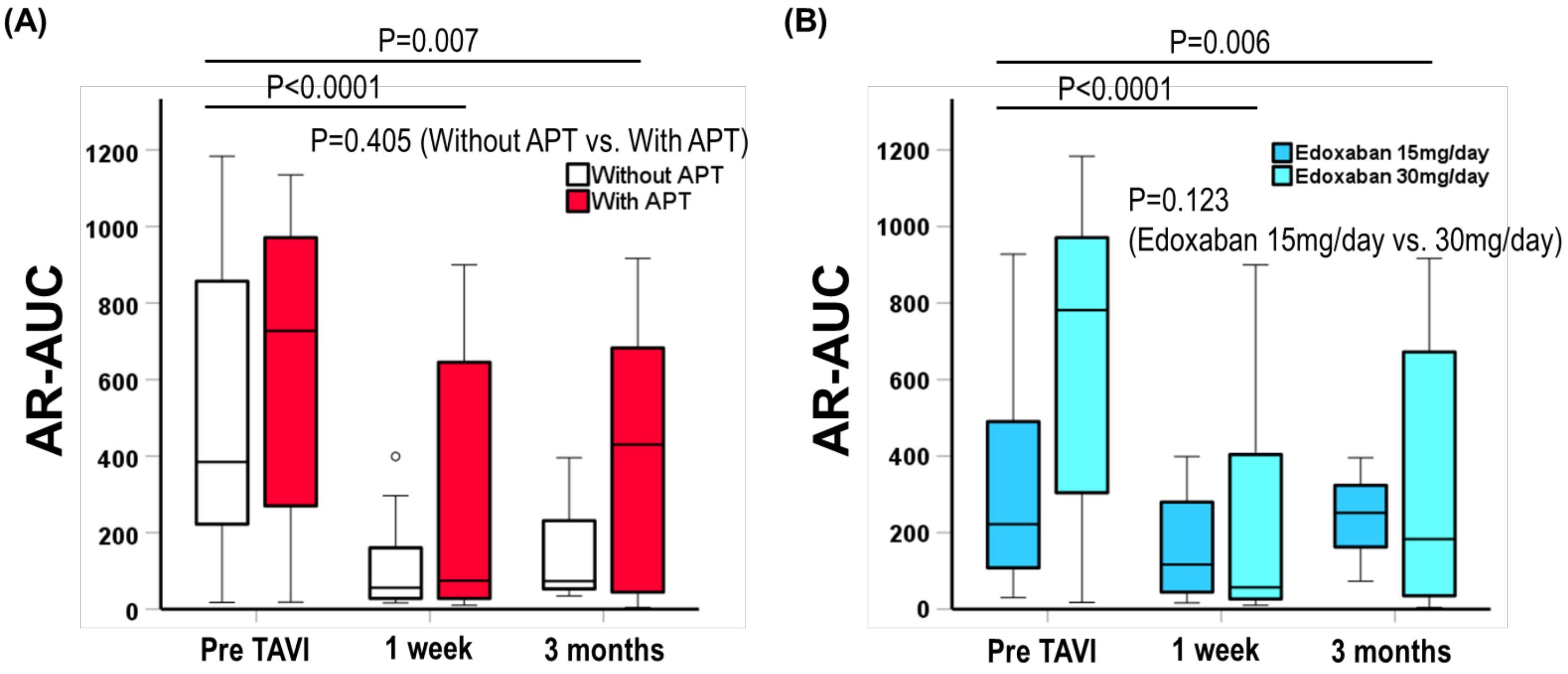

Supplementary Figure 6. Changes in thrombogenicity parameters stratified by the combination with anti-platelet therapy and different dose of edoxaban.

These figures compare the AR-AUC values before, at 1 week, and at 3 months after TAVI, among the presence or absence of APT (A) and the dosage of edoxaban (B), respectively. AR-AUC indicates area under the curve for the atheroma chip; TAVI, transcatheter aortic valve implantation; APT, antiplatelet therapy.

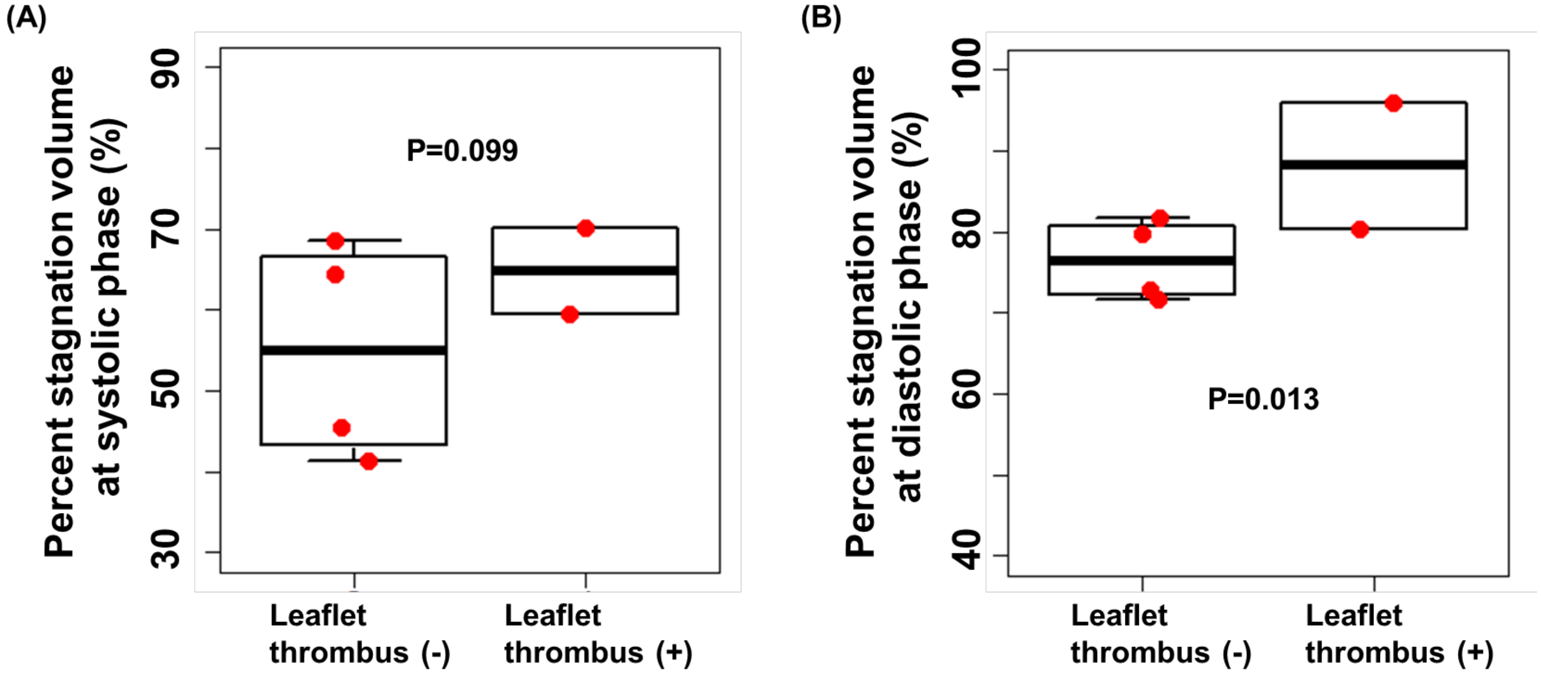

Supplementary Figure 7. Difference in flow stagnation between leaflets with and without thrombus.

Box-and-whisker plots show the difference in percent mean stagnation volume at systolic (A) and diastolic phase (B) between leaflets with and without thrombus in the patients with leaflet thrombus. The percent mean stagnation volume was calculated as the proportion of the stagnation volume within each neo-sinus and coronary cusp. To analyze the impact of percent stagnation volume on leaflet thrombus formation while accounting for individual variability, a mixed-effects model with leaflet thrombus as a fixed effect and subject-specific random effects was used.

Supplementary Table 1. Baseline characteristics of study population

Characteristics	CT assessment n = 18	Clinical outcome assessment n = 24	Characteristics	CT assessment n = 18	Clinical outcome assessment n = 24
Age, yr	84.0 (79.8, 86.0)	85.0 (79.8, 86.3)	Echocardiography		
Male	6 (38%)	10 (42%)	Ejection fraction, %	61 (53, 67)	64 (57, 68)
Body mass index	22.3 (20.8, 24.4)	21.9 (19.7, 23.8)	Aortic valve area, cm2	0.65 (0.55, 0.73)	0.62 (0.52, 0.71)
Systolic blood pressure, mmHg	114 (108, 131)	114 (109, 131)	Max V, m/s	4.32 (4.04, 4.74)	4.24 (4.00, 4.74)
Diastolic blood pressure, mmHg	69 (66, 71)	66 (65, 71)	Mean PG, mmHg	46 (38, 53)	43 (37, 51)
Symptom			Max PG, mmHg	74 (65, 90)	72 (64, 90)
Heart failure	12 (75%)	18 (75%)	AR III or IV	5 (31%)	6 (25%)
Angina pectoris	1 (6.3%)	2 (8.3%)	MR III or IV	3 (19%)	7 (29%)
Syncope	1 (6.3%)	3 (12%)	Laboratory data		
Palpitation due to AF	3 (19%)	5 (21%)	AR-AUC	501 (311, 915)	534 (274, 936)
Other	3 (19%)	4 (17%)	PL-AUC	83 (11, 241)	62 (9, 241)
Hypertension, n (%)	14 (88%)	20 (83%)	Hemoglobin, g/dL	12.20 (10.45, 12.55)	11.40 (9.75, 12.80)
Diabetes mellitus, n (%)	2 (13%)	3 (12%)	Hematocrit, %	36.3 (31.3, 39.0)	34.6 (30.8, 38.7)
Dyslipidemia, n (%)	6 (38%)	8 (33%)	Platelet count, /µL	192,000 (161,000, 199,000)	174,000 (154,000, 196,500)
Congestive heart failure, n (%)	8 (50%)	12 (50%)	BUN, mg/dL	18.8 (17.3, 24.1)	19 (18, 25)
NYHA class III or IV	3 (19%)	6 (25%)	Creatinine, mg/dL	1.03 (0.77, 1.19)	1.10 (0.92, 1.25)
History of stroke, n (%)	1 (6.3%)	4 (17%)	Creatinine clearance, mL/min	39 (32, 47)	36 (24, 44)
History of CAD, n (%)	5 (31%)	7 (29%)	T-Bil, mg/dL	0.70 (0.60, 0.90)	0.70 (0.60, 0.90)
$CHADS_2$ score	3 (2, 3)	3 (2, 3)	AST, U/L	22 (16, 28)	22 (17, 26)
CHA_2DS_2VASc score	4.5 (4, 5)	5 (4, 5)	ALT, U/L	16 (10, 24)	12.0 (9.0, 22.0)
EUROII risk score	3.98 (2.90, 4.79)	3.92 (2.82, 5.21)	PT-INR	1.22 (1.09, 1.48)	1.22 (1.10, 1.49)
STS risk score	6.3 (5.0, 7.8)	6 (4, 10)	APTT, sec	33.2 (30.3, 37.2)	33.1 (30.0, 37.1)
Clinical Frailty Scale	4 (3, 4.25)	4 (3, 4)	D-dimer, µg/mL	0.80 (0.55, 1.75)	0.80 (0.58, 1.73)
Medications			Glucose, mg/dL	104 (94, 119)	104 (95, 125)
Edoxaban dose			HbA1c, %	5.60 (5.53, 6.08)	5.60 (5.40, 6.10)
60 mg/day	1 (6.3%)	1 (4.2%)	Total cholesterol, mg/dL	181 (145, 207)	164 (146, 200)
30 mg/day	13 (81%)	18 (75%)	TG, mg/dL	100 (72, 132)	102 (71, 139)
15 mg/day	4 (22%)	5 (21%)	HDL-C, mg/dL	54 (37, 66)	54 (44, 64)
Antiplatelet therapy			LDL-C, mg/dL	114 (73, 132)	91 (72, 124)
Aspirin	10 (63%)	12 (50%)	WBC, /µL	5,400 (4,700, 6,350)	5,200 (3,908, 6,150)
Clopidogrel	3 (19%)	3 (12%)	CRP, mg/dL	0.10 (0.06, 0.45)	0.09 (0.05, 0.28)
Prasugrel	0 (0%)	0 (0%)	BNP, pg/mL	172 (86, 359)	181 (84, 312)
Other antiplatelets	0 (0%)	0 (0%)	Valve type		
ACE inhibitor	3 (19%)	3 (12%)	Balloon-expandable	12 (75%)	20 (83%)
ARB	6 (38%)	8 (33%)	Self-expanding	6 (25%)	4 (17%)

ARNI	0 (0%)	2 (8.3%)	Valve size		
Beta blockers	7 (47%)	10 (43%)	20 mm	1 (6.3%)	1 (4.2%)
Calcium channel blockers	9 (56%)	15 (62%)	23 mm	7 (44%)	12 (50%)
SGLT2 inhibitor	1 (6.3%)	2 (8.3%)	26 mm	6 (38%)	9 (38%)
Diuretics	9 (56%)	15 (62%)	29 mm	2 (13%)	2 (8.3%)
Statins	8 (50%)	10 (42%)			
Proton pump inhibitor	9 (60%)	13 (57%)			

Data are median (25%, 75%), or n (%).

AF indicates atrial fibrillation; CAD, coronary artery disease; ACE, angiotensin converting enzyme; ARB, angiotensin II receptor blocker; ARNI, angiotensin receptor neprilysin inhibitor; SGLT, sodium glucose transporter; pressure gradient; AR-AUC, area under the curve for the atheroma chip; PL-AUC, area under the curve for the platelet chip.

Supplementary Table 2: Baseline characteristics of patients with Edoxaban 15mg and 30/60mg

	Edoxaban 30 or 60 mg<br>N = 19	Edoxaban 15 mg<br>N = 5	Standardized mean differences	95% CI
Age, yr	84.0 (79.0, 86.0)	89.0 (87.0, 93.0)	-6.2	-12, -0.13
Male, n (%)	8 (42%)	2 (40%)	2.10%	-48%, 53%
Body mass index	22.3 (20.7, 23.9)	20.2 (17.5, 22.4)	1.9	-2.2, 6.0
Hypertension, n (%)	17 (89%)	3 (60%)	29%	-28%, 87%
Diabetes mellitus, n (%)	3 (16%)	0 (0%)	16%	-13%, 45%
Dyslipidemia, n (%)	6 (32%)	2 (40%)	-8.40%	-65%, 48%
Congestive heart failure, n (%)	9 (47%)	3 (60%)	-13%	-74%, 48%
History of stroke, n (%)	4 (21%)	0 (0%)	21%	-9.9%, 52%
History of coronary artery disease, n (%)	6 (32%)	1 (20%)	12%	-41%, 64%
Ejection fraction, %	66 (58, 70)	58 (52, 59)	6.8	-3.5, 17
EURO II risk score	3.83 (2.65, 4.67)	8.00 (7.45, 8.55)	-4	-12, 4.1
STS risk score	5 (4, 7)	25 (20, 25)	-14	-28, -1.0
Clinical Frailty Scale	4 (3, 4.5)	4 (3, 4.5)	0.16	-0.83, 1.1
$CHADS_2$ score	3 (2, 3)	2 (1.5, 3)	0.73	-0.27, 1.7
CHA_2DS_2VASc score	5 (4, 5)	4 (3, 5)	0.71	-0.29, 1.7
Laboratory data				
AR-AUC	781 (343, 971)	222 (108, 490)	308	-136, 752
PL-AUC	64 (10, 248)	7 (5, 93)	-47	-460, 367
Hemoglobin, g/dL	12.25 (10.45, 12.98)	9.40 (9.20, 10.30)	2.2	1.1, 3.3
Platelet count, /µL	193,000 (165,000, 199,500)	134,000 (102,000, 155,000)	59,578	3,495, 115,660
BUN, mg/dL	20 (17, 26)	19 (18, 25)	-5.4	-28, 17
Creatinine, mg/dL	1.03 (0.90, 1.20)	1.33 (1.26, 1.47)	-0.28	-0.75, 0.18
Creatinine clearance, mL/min	37 (29, 46)	21 (18, 25)	14	-0.13, 28
PT-INR	1.22 (1.10, 1.57)	1.20 (1.13, 1.29)	0.11	-0.16, 0.38
APTT, sec	33.1 (30.0, 37.9)	32.0 (30.3, 34.4)	1.1	-4.1, 6.3
D-dimer, µg/mL	0.80 (0.60, 1.70)	1.40 (0.90, 1.60)	0.43	-1.0, 1.9
Antiplatelet therapy				
Aspirin	12 (63%)	0 (0%)	63%	29%, 97%
clopidogrel	3 (16%)	0 (0%)	16%	-13%, 45%
prasugrel	0 (0%)	0 (0%)	0.00%	0.00%, 0.00%
Other antiplatelet	0 (0%)	0 (0%)	0.00%	0.00%, 0.00%

Data are median (25%-75%), or n (%).

AR-AUC indicates area under the curve for the atheroma chip; PL-AUC, area under the curve for the platelet chip.

Supplementary Table 3: Clinical outcomes in 24 patients

	1 week	3 months	Total
All-cause death	0 (0%)	0 (0%)	0 (0%)
Cardiovascular death	0 (0%)	0 (0%)	0 (0%)
Non-cardiovascular death	0 (0%)	0 (0%)	0 (0%)
Acute myocardial infarction	0 (0%)	0 (0%)	0 (0%)
Cerebrovascular event	0 (0%)	0 (0%)	0 (0%)
Ischemic stroke	1 (4.2%)	1 (4.2%)	2 (8.3%)
TIA	0 (0%)	0 (0%)	0 (0%)
Acute kidney injury	0 (0%)	0 (0%)	0 (0%)
Bleeding events	3 (12.5%)	0 (0%)	3 (12.5%)
Life-threatening or disabling bleeding	2 (8.3%)	0 (0%)	2 (8.3%)
Major bleeding	0 (0%)	0 (0%)	0 (0%)
Minor bleeding	1 (4.2%)	0 (0%)	1 (4.2%)
New conduction disorders	0 (0%)	0 (0%)	0 (0%)

Data are n (%).

TIA indicates transient ischemic attack.

Supplementary References

1. Ruggeri ZM, Zimmerman TS. The complex multimeric composition of factor viii/von willebrand factor. *Blood*. 1981;57:1140-1143
2. Tamura T, Horiuchi H, Imai M, Tada T, Shiomi H, Kuroda M, Nishimura S, Takahashi Y, Yoshikawa Y, Tsujimura A, Amano M, Hayama Y, Imamura S, Onishi N, Tamaki Y, Enomoto S, Miyake M, Kondo H, Kaitani K, Izumi C, Kimura T, Nakagawa Y. Unexpectedly high prevalence of acquired von willebrand syndrome in patients with severe aortic stenosis as evaluated with a novel large multimer index. *J Atheroscler Thromb*. 2015;22:1115-1123
3. Horiuchi H, Doman T, Kokame K, Saiki Y, Matsumoto M. Acquired von willebrand syndrome associated with cardiovascular diseases. *J Atheroscler Thromb*. 2019;26:303-314
4. Itatani K, Miyaji K, Qian Y, Liu JL, Miyakoshi T, Murakami A, Ono M, Umezu M. Influence of surgical arch reconstruction methods on single ventricle workload in the norwood procedure. *J Thorac Cardiovasc Surg*. 2012;144:130-138
5. Numata S, Itatani K, Kanda K, Doi K, Yamazaki S, Morimoto K, Manabe K, Ikemoto K, Yaku H. Blood flow analysis of the aortic arch using computational fluid dynamics. *Eur J Cardiothorac Surg*. 2016;49:1578-1585
6. Miyazaki S, Itatani K, Furusawa T, Nishino T, Sugiyama M, Takehara Y, Yasukochi S. Validation of numerical simulation methods in aortic arch using 4d flow mri. *Heart Vessels*. 2017;32:1032-1044
7. Ishii M, Kaikita K, Mitsuse T, Nakanishi N, Oimatsu Y, Yamashita T, Nagamatsu S, Tabata N, Fujisue K, Sueta D, Takashio S, Arima Y, Sakamoto K, Yamamoto E, Tsujita K. Reduction in thrombogenic activity and thrombocytopenia after transcatheter aortic valve implantation - the attractive-ttas study. *Int J Cardiol Heart Vasc*. 2019;23:100346
8. Granegger M, Thamsen B, Hubmann EJ, Choi Y, Beck D, Valsangiacomo Buechel E, Voutat M, Schweiger M, Meboldt M, Hübler M. A long-term mechanical cavopulmonary support device for patients with Fontan circulation. Med Eng Phys. 2019 Aug;70:9-18.
9. Itatani K, Miyaji K, Ohara K, Ishii M. Computational Fluid Dynamic Simulations on Fontan Circulation. Pediatric Cardiology and Cardiac Surgery 2010, 26(1), 39-48. (in Japanese)
10. Ruile P, Jander N, Blanke P, Schoechlin S, Reinöhl J, Gick M, Rothe J, Langer M, Leipsic J, Buettner HJ, Neumann FJ, Pache G. Course of early subclinical leaflet thrombosis after transcatheter aortic valve implantation with or without oral anticoagulation. *Clin Res Cardiol*. 2017;106:85-95
